# The Poor Lady

**Published:** 1901-05-04

**Authors:** 


### The Hospital

A Journal of

The Medical Sciences and Hospital Administration.

Vol. XXX.—No. 762] Edited by Sir Henry Burdett, K.C.B., and Solomon C. Smith, M.D., M.R.C.P. [May 4, 1901.

### The Poor Lady.

Attention has lately been called in the columns of more than one of our contemporaries, and especially by letters which have been published in the *Times*, to the hard lot of that very widely diffused member of society whose position has been briefly described as that of "a poor lady." The phrase derives comprehensiveness from its very ambiguity; and this comprehensiveness is unfortunately necessary in order to render it capable of embracing even a small portion of those to whom it is intended to apply. To compose a generic description of the "poor lady" would be a task calculated to present difficulties even to such a genius as that of Mrs. Gaskell or of George Eliot; but there are few readers who have not encountered, or, indeed, who do not almost daily encounter, what may be described as concrete examples of the class. As existing at the present day, she is usually of what Lord Byron called "a certain age"—that is, she has come down to us from a period when the education of women, even of the better classes, was of an exceedingly superficial kind, directed chiefly towards an acquaintance with the duties of wifehood, of mistress-ship, and of maternity, when what Americans are said to describe as "parlour tricks" were regarded as essential to the full development of young ladyhood; when little tunes were feebly played, when little ballads were feebly quavered, when the "dignity of labour" was an unknown, or at least an unappreciated phrase, and when schoolmistresses would have been shocked at any appearance of self-reliance among their pupils. Under such a system,

> "To hear, to heed, to wed—
> Fair lot that maidens choose,"

was regarded as the natural destiny of women; and the inexorable census had not yet brought home to the minds of the majority that there were some hundreds of thousands of them by whom, on account of arithmetical considerations alone, this destiny could never be fulfilled. The duty of making provision for daughters, which forms so prominent a feature of the social life and domestic polity of France, has never, in this country, been sufficiently recognised and acted upon; and the general result is that, at the opening of the twentieth century, we find multitudes of women who are no longer young, who are raised above sordid poverty, but not to the level of reasonable comfort, and whose colourless lives are spent in a ceaseless effort to make sixpence do the work of a shilling, and to cheat both themselves and those about them into the belief that they have no inclination for pleasures or for luxuries, however inexpensive or enjoyable, which their want of means compels them to forego. The strain is hard enough in health, but in illness it attains the breaking point. To the honour of the medical profession it may be said that patients of this class constantly receive an amount of sympathetic care which wealth could not command, and it may be hoped that very few of them are absolutely friendless. But the cheap lodging is not very accessible; the friends are more or less caught up and carried round in the vortex of life, or, more pitiful still, they pass away and there are none to take their places, while the "poor lady" remains. Her very poverty implies conditions of living which do not appear to be antagonistic to length of days; and so it befalls that she, to whom death would not seldom come as a relief from daily care, is left as the survivor of those who would once have cheered her solitude or smoothed her pillow. Against maladies of a serious kind, as was pointed out by one of the writers to the *Times*, resources are furnished by such institutions as the invalid homes for gentlewomen; but the accommodation provided by these is infinitely less than the demand, and the more urgent need is for homes in health rather than for mere places of resort in sickness.

It must be considered that the claims of the "poor lady," as a member of a class, have arisen not so much from neglect on the part of the last generation as from the development of a feverishness of competition which parents in that generation could hardly be expected to anticipate, and that these claims must therefore be regarded as making legitimate demands upon the charity of the present day, and especially upon that better type of charity which is displayed by gifts of guidance rather than by gifts of money.

England is emphatically the country of the "great lady," and the great lady could at present undertake no more noble work than that of establishing an organisation for the assistance of her "poor" sister—an organisation for rescuing her, by the agency of co-operative homes, from the tyranny of grasping and dishonest landladies, for enabling her slender funds, by the aid of judicious combination, to buy in the cheapest instead of in the dearest markets, and for assisting her to place in security any savings which improved conditions might render it possible for her to make. In the present day, the state of public opinion with regard to female industry and independence has undergone what is less a change than a revolution; and the opportunities and varieties of employment now opening before women are so numerous as to afford reasonable ground for hope that, when this century is passing away, poor ladies will at least be no more plentiful than are poor gentlemen among ourselves. Some, of course, there will always be; because there must always be, in both sexes, some who are unable to adjust themselves to their environment, and so to make the greatest use of whatever advantages it may afford. At present, the need is not only great, but pressing; and the victims, from the very circumstances of their condition, are powerless to make their wants heard above the din by which they are surrounded. Any possible definition of the class implies that the ladies, although "poor," are not "destitute," and that they have means which, although separately inadequate, might be rendered effective by combination. The case is not one for almsgiving, but for leadership; and it will be a lasting discredit to the women of England if no competent leader can be found among their ranks.

